# Effect of Arbuscular Mycorrhizal Fungi Isolated From Rock Phosphate Mine and Agricultural Soil on the Improvement of Wheat Plant Growth

**DOI:** 10.3389/fmicb.2022.881442

**Published:** 2022-05-25

**Authors:** Zakaria Hazzoumi, Salah Eddine Azaroual, Najib El Mernissi, Youssef Zaroual, Robin Duponnois, Brahim Bouizgarne, Issam Meftah Kadmiri

**Affiliations:** ^1^Green Biotechnology Laboratory, Moroccan Foundation for Advanced Science, Innovation and Research, Rabat, Morocco; ^2^Laboratory of Plant Biotechnology, Team of Microbial Biotechnology, Faculty of Sciences, Université Ibn Zohr (UIZ), Agadir, Morocco; ^3^OCP/Situation Innovation, OCP Group, Jorf Lasfar Industrial Complex, El Jadida, Morocco; ^4^Laboratory of Tropical Symbioses and Mediterranean, Environment and Resources Department, Institute of Research for Development, Marseille, France; ^5^Plant and Soil Microbiome Sub-Program, Biodiversity and Plant Sciences, AgroBioSciences, Mohammed VI Polytechnic University (UM6P), Ben Guerir, Morocco

**Keywords:** AMF, rock phosphate, triple superphosphate (TSP), plant growth, glomus, gigaspora, wheat plant, phosphate uptake

## Abstract

The improvement of plant growth and yield becomes crucial to feed the rising world population, especially in harsh conditions, drought, salt stress, lack of nutrition, and many other challenges. To cope with these stresses, plants developed an adaptation strategy (mycorrhiza), which is an efficient way to reinforce their growth and resistance. For this purpose, we studied the influence of mycorrhizal fungi isolated from a natural rock phosphate mine in the vicinity of some native plants and agricultural soil to assess their capacity in increasing the growth, nutritional profile improvement, and biochemical parameters in the inoculated wheat plants. Results showed a high diversity of isolated arbuscular mycorrhizal fungi (AMF) spores in the agricultural soil, and less diversity in the natural phosphate samples, where three main genera were identified: glomus, gigaspora, and acaulospora. The chlorophyll content increased by 116% in the native inoculum (NM) flowed by Glomus sp2 from agricultural soil (98%) compared to non-mycorrhized plants, which significantly impact the growth and plant biomass (an increase of 90 and 73%, respectively). The same rate of change was shown on total phenolic compounds with an increase of 64% in the plants inoculated with Glomus sp2 in the presence of TSP, compared to the non-mycorrhized plants. In conclusion, the inoculation of wheat plants with AMF spores improved plants’ growth *via* the increase in the density of the root system, which implies better assimilation of nutrients, especially in mycorrhizal plants with phosphorus fertilization regime, triple superphosphate (TSP) or natural rock phosphate (RP). This improvement of the physiological and biochemical parameters (chlorophyll contents and phenolic compound) of the treated plants reflected the positive impact of AMF, especially those originating from RP. AMF in phosphate mine could be an important source of inoculum to improve plant nutrient efficiency with the direct use of RP as fertilizer.

## Introduction

Phosphorus (P) is the second significant element required for plants growth and development (Razaq et al., 2017). With nitrogen (N) and potassium (K), P is considered as one of the three macronutrients necessary for plant growth and development, and is generally applied to soils through chemical fertilizers (Liu et al., 2014). P is an essential element of the energy metabolism, living cells since it is the main component of DNA, RNA, ATP, and phospholipids (the major component of cell membranes). It is, therefore, involved in cell division, the transmission of genetic information, the transfer and storage of energy (ATP), and also in the photosynthetic system. Plants take up P from the soil as orthophosphate form (Pi); however, the concentration of this latter in the soil rarely exceeds 10 mM (Daniel et al., 1998). Although the total P content (inorganic and organic fractions) of soils generally does not exceed 0.12%, only 0.1% of the total P exists in the soluble inorganic form, easily assimilated by plants (Goldstein, 1994). On the other hand, a very important amount of insoluble phosphate exists in different forms, such as dicalcium phosphate, tricalcium phosphate, rock phosphate, and hydroxyapatite.

Morocco, China, and the United States are the three main producers of rock phosphate in the world. They hold more than 80% of the exploitable deposits. This rock phosphate presents a good opportunity to be applied directly to the soils and considered as a cheaper fertilizer, affordable for low-income countries. However, there is a need for a smart way to convert insoluble rock phosphate to soluble form, easily available for the plants. The use of microorganisms presents one of the innovative and challenging approaches to RP solubilization. Microorganisms produce phosphatase enzymes (Stephen et al., 2015) and organic acids (Reyes et al., 2006). The latter chelate the cations attached to insoluble phosphates through their carboxylic groups, allowing them to be converted into soluble forms (Mackey and Paytan, 2009). These microorganisms directly benefit from the bioavailable P necessary for their growth (Azaroual et al., 2020). Likewise, other organisms are able to take advantage of solubilized P, such as symbiotic association between fungi and plants, largely named by mycorrhization, which is considered as one of the oldest associations between plants and microorganisms on earth. This association allows the both partners to grow, explore water and nutritional resources, and adapt to several kinds of environment and harsh conditions (Hazzoumi et al., 2015; Damin et al., 2020). Up to 80% of terrestrial plants species, including wild plants, trees, common and economically valuable crops, are estimated to be colonized by AMF (Tarkka and Frey-Klett, 2008).

One of the first benefits of AMF to the plants is the exploitation of water and nutrients, thanks to a very developed root system constituted principally by a huge hyphal network that connects plant interior to surrounding soil. Thus, hyphae are more extensive and more efficient than root hairs in taking up nutrients (Hazzoumi et al., 2017). However, plants’ nutritional profiles are mainly depending on macronutrients, mainly nitrogen and phosphorus (P). When soil is poor, AMF provide P and other micronutrients poorly available in the medium to the plants (Kamrun et al., 2020). Mutual beneficial association consists of supplying carbon (C) to the fungus from the plants and the P, microelements, and water by the fungus to the plants (Smith et al., 2003; Hazzoumi et al., 2017; Kamrun et al., 2020).

This association highly depends on the plant species, soil microorganisms (mycorrhiza and helping bacteria), disease, and environmental conditions. Under harsh conditions, Mycorrhizal associations help plants to ensure a better balance of nutrition uptake and tolerance to biotic and abiotic stresses (Damin et al., 2020; Ranjbar Sistani et al., 2020). According to some authors, the rates of root colonization and spores production by *Glomus fasciculatum* and *Acaulosporalaevis* in *Sesamum indicum* plants were highest in soil with increased concentration of Rock Phosphate (up to 22.5 mg/kg) (Sabannavar and Lakshman, 2009). On other hand, Kamrun et al. (2020) showed that the colonization may only be a suitable trait for improved P efficiency in farming systems where available P is consistently low. According to this study, P fertilizers reduced AMF colonization in wheat variety *Carazinho* and *RAC875* that has a low-yield response to P fertilization.

The kind of fertilizer used and the concentration of free P in soil significantly impact mycorrhization efficiency. Kuramshina et al. (2020) investigated the effect of Gumi-Omi-Phosphorus organic fertilizer and the superphosphate mineral fertilizer on the biomass of roots and the formation of arbuscular mycorrhiza on *Pisum sativum* L., *Avena sativa* L., and *Triticum aestivum* L. Their results showed that, with the use of fertilizers, root biomass increased in all studied plants. At the opposite, the colonization intensity decreased when superphosphate was introduced into the soil. In unsuitable soil, the Gumi-Omi-Phosphorus could stimulate both partners to form an arbuscular mycorrhizal symbiosis. The unlikable impact of superphosphate is probably due to the presence of readily available phosphorus in greater quantities in the soil. Hence, better and efficient mycorrhization on the plants has been related to the rate of nutrients mobilization from soil to the plants (Bolan, 1991; Kobae, 2019) and helps this later to explore, accumulate, and transport the free phosphate (Pi). This fast movement of nutrients, mainly (P) into mycorrhizal hyphae, is achieved by increasing the affinity of this latter and by decreasing the threshold concentration required for the P absorption. However, in the plant colonized by many AMF, the ability of this latter regarding phosphate uptake differs (Kobae, 2019). Mycorrhization efficiency is also related to the dynamism of AMF, and, until now, this process is still poorly investigated and remained unknown. The different responses of the plants to AMF were mainly due to the high intraspecific variability in the fungal gene repertoires, which presents a major factor that explains this affinity between plants and fungi (Chen et al., 2018). In addition, the process by which AMF transform the organic phosphate (Po) to inorganic one (Pi) is named fine-tuning of phosphate homeostasis (PHO), which implicates the expression of PHO genes, including those encoding Pi transporters and the extracellular A. According to Saito and Ezawa (2016) and Tatsuhiro and Katsuharu (2018), uptake processes of Pi from soil to fungi implicate H^+^/Pi symporters and Na^+^/Pi symporters, driven by H^+^ATPases and Na^+^ATPases, respectively. Through these processes, the fungi are capable to accumulate a massive amount of polyP (up to 64% of total cellular phosphorus) (Tatsuhiro and Katsuharu, 2018); such a process is strictly regulated in the fungal cells. Another mechanism is consisting the mineralization of Po by its extracellular acid phosphatases (ACPs) (Koide and Kabir, 2000). Bulgarelli et al. (2020) explained that roots acquire phosphorus (P) as orthophosphate (Pi) through phosphate transporters of the PHT1 family with different affinities to Pi; this process is significantly influenced by arbuscular mycorrhizal (AM) symbiosis.

Generally, these microorganisms (AMF in association with bacteria) release three groups of enzymes able to mineralize P from organic amalgams in the soil environment (Azaroual et al., 2020; Etesami et al., 2021). The first group consisted on phytases that precisely cause phosphorus discharge from phytic acid; the second is related to non-specific phosphatases that accomplish dephosphorylation of phosphoester and phosphoric anhydride bonds in organic stuff, and, finally, phosphonatases and C–P lyases, enzymes that achieve carbon–phosphorus cleavage in organophosphonates (Lusk et al., 2017; Gong et al., 2018; Kour et al., 2020; Patel and Goswami, 2020; Rastegari et al., 2020).

In this work, we aimed to study and explore the diversity and the application of AMF isolated from two different environments: Moroccan phosphate mines and agricultural soil. Also, the ability of isolated AMF to improve the growth and nutrition by using different sources of phosphate (RP & TSP), their effects of the growth performance, and the physiological parameters of wheat plants were also investigated.

## Materials and Methods

### Samples Collection

#### Collection of Phosphate Mines Native Plants and Bulk Soils

Three plant species, which grow spontaneously in the phosphate mine of Ben Guerir (the Rhamna region, the center of Morocco), mainly in the humid season, were selected. They were identified as *Digitariasanguinalis*, *Solanum americanum*, and *Alternanthera caracasana*. The plants were picked up from their natural environment (phosphate mine), and their roots were analyzed for the presence of AMF.

#### Estimation of Mycorrhization in Rock Phosphate Native Plants

The roots were stained according to the method described by Derkowska et al. (2015). Microscopic specimens were prepared and examined with a microscope (Nikon 50i) using objectives with magnifications of ×20, ×40, ×60, ×100. The assessment of the colonization degree of the roots by arbuscular mycorrhizal fungi was performed with the Trouvelot method (Trouvelot et al., 1986). This method is based on the assessment of mycorrhizal frequency (F%) and relative mycorrhizal intensity (M%) that are calculated according to the formula described by Mikiciuk et al. (2019).

#### Collection of Agricultural Soils

Agricultural soil samples were collected from two regions in the vicinity of Settat Town (70 km in the south of Casablanca): the first zone is Douar Boukallou, Latitude: 33.0319; Longitude: −7.6202, and the second zone is Ouled Said, Latitude: 33,019; Longitude: −7.70.

A total of five different soil samples belonging to the two different habitats were used for the AMF spores isolation and investigation as presented in Table 1.

**TABLE 1:** Description of samples used for AMF isolation and investigation.

Sample type	Sample name	Location	Assay types
Phosphate mine plants	*Digitaria sanguinalis* rhizosphere	Ben Guerir Phosphate mine	Investigation of root mycorhization
	*Solanum americanum* rhizosphere	Ben Guerir Phosphate mine	Investigation of root mycorhization
	*Alternanthera caracasana* rhizosphere	Ben Guerir Phosphate mine	Investigation of root mycorhization
	*Bulk soil of the phosphate mine*	Ben Guerir Phosphate mine	Mycorrhizae isolation
Agricultural soil	Soil Zone 1	Douar Boukallou, Settat region	Mycorrhizae isolation
Agricultural soil	Soil Zone 2	Ouled Said Latitude, Settat region	Mycorrhizae isolation

#### Arbuscular Mycorrhizal Fungi Spores, Isolation, and Identification

Arbuscular mycorrhizal fungi spores were isolated from the two different regions: the agricultural area (the Settat region) and the rock phosphate mines (the BenGuerir region) (Table 1). Each sample consisted of five 100 g sub-samples collected at 20-cm depth. The soil was carefully mixed, and spores of AM fungi were extracted from each sub-sample (100 g) by wet sieving and decanting technique, followed by sucrose centrifugation (Sieverding, 1991). The supernatant was poured through a 40-mm sieve and rinsed with tap water. Fungal spores were surface sterilized with a chloramine T solution (0.2 g L^–1^) and streptomycin (0.2 g L^–1^) (Mosse, 1973) in order to eliminate the mycorrhizosphere microflora. The spores were examined with a binocular magnifying glass or microscope; then, they were kept in distilled water at 4°C for 2 days before use.

#### Preparation and Multiplication of Inoculum From Isolated Spores

In this study, four morphotypes were used for inoculum preparation. Three morphotypes were sampled from agricultural soils and named *Glomus* sp.1 (G. sp1), *Glomus* sp.2 (G. sp2), and *Acaulosporalongula* (A. long). One other morphotype named Morphotype MN (MN) was selected from phosphate mine samples.

Barley plants *(Hordeum vulgare)* were used for AMF multiplication (spores isolated from phosphate mine and agricultural soil). Barley plants are characterized by very significant ramification of the root system. The latter is fibrous and extensive, which increases the number of potentially colonizable sites but fragile and requires delicacy in the revelation and the realization of the assembly. This cereal has significant compatibility with a wide range of mycorrhizal genera (Chaurasia and Khare, 2006). Fragments of roots of inoculated barley plants, hyphae, and propagules were mixed with the growing medium (the soil) at a ratio of 5 g/400 g of soil (w/w). The positive control plants were inoculated with a reference inoculum *Glomus intraradices* (MG).

### Plant Material and Growth Conditions

#### Wheat Seeds Germination and Inoculation Assays

Wheat seeds (*Triticum aestivum*) were disinfected by ethanol 90° for 30 s to 1 min, and then by a solution of mercury hypochlorite (1%) for 3–4 min. Disinfected seeds were soaked in sterile water and placed in Petri dishes to germinate in the dark at a temperature of 26°C in an incubator. After germination, the seedlings were transplanted into the growth medium (mixture between peat and soil 2/1) and despatched in six categories, non-mycorrhized plants (NM without TSP, neither RP), non-mycorrhized + RP, non-mycorrhized + TSP, mycorrhized (without TSP, neither RP), mycorrhized + TSP, and mycorrhized + RP. RP and TSP were used in this study as two different P fertilization regimes, with 45 μg of RP/g of soil and 15-μg TSP/g of the soil, respectively, in order to assess the effect of the mycorrhizal association on the growth and of phosphate uptake.

The inocula used in these experiments were mycorrhizal spores isolated, identified according to their morphotypes and multiplied within the green biotechnology laboratory at MAScIR Foundation as previously described. Each morphotype was used separately to inoculate the plant.

#### Growth Conditions and Assessment Parameters of Plant Inoculation by Mycorrhiza

The experimental design consisted of five replications in pots, containing the previous mixture (each spore morphotype isolated from agricultural or phosphate mine, RP, TSP with wheat plants) and two controls: the reference inoculum *Glomus intraradices* (GM) (positive control) and without inoculation (negative control) arranged in randomized block design. The pots were then placed in phytotron at 26°C, photoperiod of 16:8 h with an illumination intensity of 240 μmol m^–2^ s^–1^. After 8 weeks, the wheat plants were collected for mycorrhizal richness evaluation and for physiological and biochemical parameters determination.

#### Determination of Chlorophyll Content

Leaf fragments (1 g) were grounded in a mortar previously placed in ice with a pinch of magnesium carbonate and 5 g of anhydrous sodium sulfate in 10 ml of 80% acetone. After filtration, the residue was recovered in test tubes. Other extractions were carried out with acetone until a colorless filtrate was obtained (devoid of any traces of chlorophyll pigments). The different filtrates were combined, and the final volume was noted (Inskeep and Bloom, 1985). Optical densities (ODs) were measured at 645 and 663 nm for the chlorophylls *a* and *b*, respectively, using a spectrophotometer (Ultrospec 2100 Pro, The Amersham Biosciences), and the concentrations were determined according to the following formula:

Chlorophyll *a* = (0.0127 OD_663_) – (0.00269 OD _645_)

Chlorophyll *b* = (0.0229 OD _645_) – (0.00468 OD _663_)

Total chlorophyll = (0.0202 OD _645_) + (0.00802 OD _663_)

#### Extraction and Determination of Total Phenols

Fragments of leaves and roots (0.5 g) were grounded in a mortar, containing 5 ml of ethanol, 50% (water-alcohol solution). Then, the extracts were collected in tubes with lids and numbered. The tubes were then kept at 4^°^C overnight to allow ethanol to extract the maximum amount of phenolic compounds present in the extract.

In the tubes containing the leaf extracts, 0.5 ml of chloroforms was added to 3 ml of extract, and the tubes were vortexed and centrifuged 5 min at 5,000 rpm; the two phases supernatant and pellet were separated (Ribereau-Gayon and Stonestreet, 1966). Total phenolic compounds determination was performed by using Folin-Ciocalteu reagent (Ribereau-Gayon and Stonestreet, 1966). Briefly, the following mixture was prepared in test tubes: 0.5 ml of extract, 3 ml of water, 0.5 ml of Na_2_CO_3_ (20%). Then, 0.5 ml of Folin-Ciocalteu reagent was added. After that, the tubes were placed in an oven at 40°C for 30 min. The absorbance was read at 760 nm, and the content of phenolic compounds was calculated. Gallic acid was used for standard curve determination. Results were expressed in milligrams per gram of fresh leaf matter.

#### Quantitative Determination of NPK

Approximately 0.4 g of each plants were transferred to digestion tubes and the digestion mixture (Sulfuric acid-Selenium) was added to samples. The digestion tubes were transferred to block digester preheated to 300°C. The analysis were carried out using automated Continuous Flow Analyzer (CFA) (Skalar, Netherlands) according to the manufacturer instructions.

Ionic analysis of NPK in the plant leaves was carried out according to Novozamsky et al. (1983) methods. The P analysis was based on molybdenum blue method that lies on the reaction between orthophosphate ions with ammonium heptamolybdate and potassium antimony (III) oxide tartrate forming the antimony-phospho- molybdate complex. This latter is intensely reduced to a blue color by ascorbic acid, then the standard curve of potassium dihydrogen phosphate (KH_2_PO_4_) was prepared in the range of 0.2; 0.4; 0.6; 0.8 and 1 mg P.L^−1^ (Steele et al., 1984). The other elements were determined according to manufacturer recommendations.

### Statistical Analysis

One-way ANOVA was carried out for each parameter studied. Tukey’s *post-hoc* multiple mean comparison test was used to evaluate the significant differences between treatments (at a 5% level). Univariate analysis was used to test significant differences in treatments, accessions, and their interaction for an individual parameter. All statistical analyses were performed with IBM.SPSS statistics, Version 21. The results of each experiment (biochemical essays) were repeated three times. The principal component analysis was made by R software.

## Results

The physicochemical characteristics of the phosphate rock soils used along this work reflected an almost neutral pH (7, 83) and conductivity of 0.415 mS. Furthermore, for its mineral composition, the P content was around 51 mg/g, the N between 0.42 and 0.46 mg.g^–1^, and K with average content of 12 mg.g^–1^ (Table 2). On other hand, the agricultural Zone 1 is characterized by a slightly basic pH (8, 68) and high conductivity (35, 54); also, the two agricultural soils showed a considerable concentration of N, P, and K higher than in rock phosphate.

**TABLE 2:** NPK composition and physico-chemical property of the phosphate rock, and agricultural soil samples.

Samplings	N (mg/g^–1^)	P (mg/g^–1^)	K (mg/g^–1^)	pH	Conductivity mS
Rock phosphate	0.45 ± 0.02	51 ± 5	12.58 ± 3.8	7.83 ± 0.18	0.415 ± 0.14
Zone 1	1.27 ± 0.38	60.22 ± 15.71	191.5 ± 15.7	8.68 ± 0.3	35.54 ± 78
Zone 2	1.15 ± 0.26	165.55 ± 23.93	296.83 ± 23.93	7.71 ± 0.24	65.54 ± 46

### Spores Isolation and Observations

#### Spores Isolation From Agricultural Soil Samples

**FIGURE 1:**
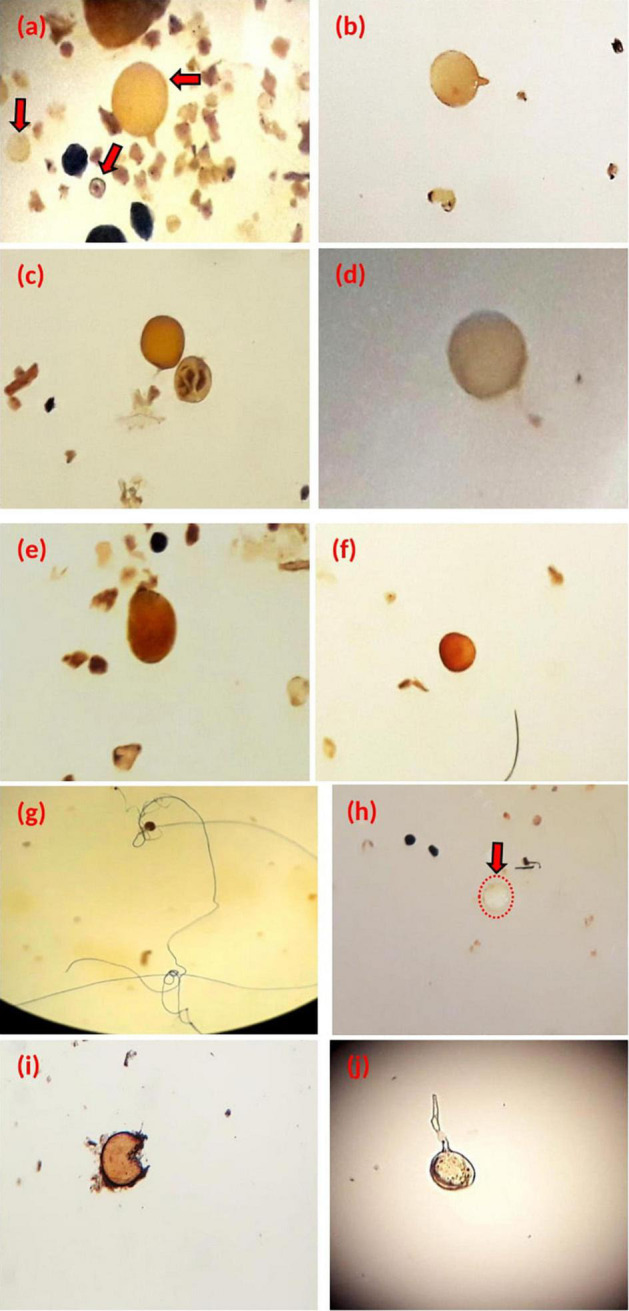
Spores of different size and colors isolated from agricultural soil samples, **(g,h)** observation under magnifying glass (×4), *Glomus sp1* **(a–c)**, (**d:** *Gigaspora sp1*), (**e:** *Gigaspora sp2*), (**f:** *Acaulospora longula*), **(g)** *Glomus sp2*, *Glomus sp3* **(h)**, observation (**i,j**: unidentified) and made under the microscope (×200).

The sieving and decanting technique revealed the presence of diversity of spore morphotypes in terms of shape, size, color, as well as the presence of conical structures at the external surface of the spores, or of the hyphae in agricultural soil samples (Figure 1).

According to their morphological criteria, isolated spores could be grouped into three genera: *Glomus* (Figures 1a–c), which is characterized by large unicellular chlamydospores, globular oval in shape, born from a single hypha. In addition, the genus *Glomus* is often characterized by sporocarp. The presence of the suspensory hypha, which connects the spore to the mycelium, is the main characteristic of this genus (Walker, 2013; Melo et al., 2020).

*Gigaspora* (Figure 1d), known as “giant spores,” are spores of large diameter and solitary, and the suspending hypha present the bulb morphology. This genus has been observed among white, white-yellow, and black spores, with an average diameter of 191, 267.5, and 441 μm, respectively (Walker, 2013; Melo et al., 2020).

*Acaulospora* (Figure 1f) spores of brown color are characterized by a thin wall formed by the apical swelling of a hypha. The spores differentiate laterally from the hyphae-carrying saccules (Khare and Arora, 2015).

Spores of the morphotype presented in Figures 1a,c,d (identified as the genus *Glomus*) were the most abundant in the agricultural soil samples. They were selected for multiplication and, also, for studying their effect on the growth and the nutritional profile of wheat plants.

The studied agricultural soil samples showed also characteristic structures (suspensory hyphae, saccules), and, sometimes, small spores of unidentified fungi that were observed, which required further studies (Figures 1i,j).

Figure 1g showed a spore with a highly developed hypha, which was the link between the root cells and the extra-root environment. The isolated spores were collected and stored for multiplication and agronomic assays.

**FIGURE 2:**
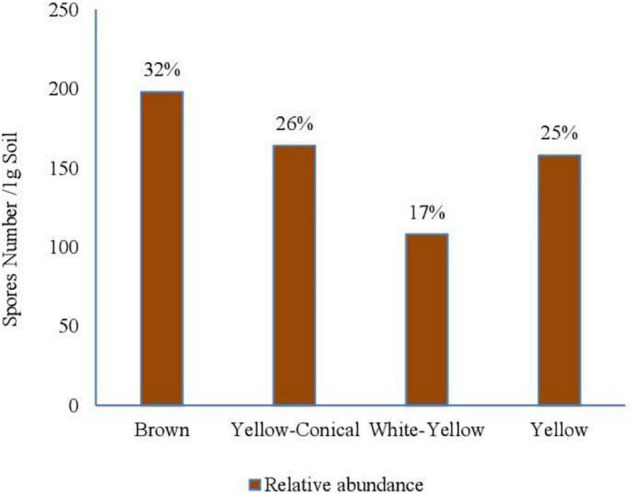
Relative abundance (%) of spore morphotypes in the agricultural soil samples.

Results of relative abundance showed that the morphotype “Brown” spores were the most abundant in agricultural soil, 32% of the total recovered spores, and then the “yellow-conical” and “yellow” spores with 26 and 25%, respectively (Figure 2). Finally, the “white-yellow” spores represented relative abundance of 17% in these samples.

#### Spores Isolation From Phosphate Mine Samples

**FIGURE 3:**
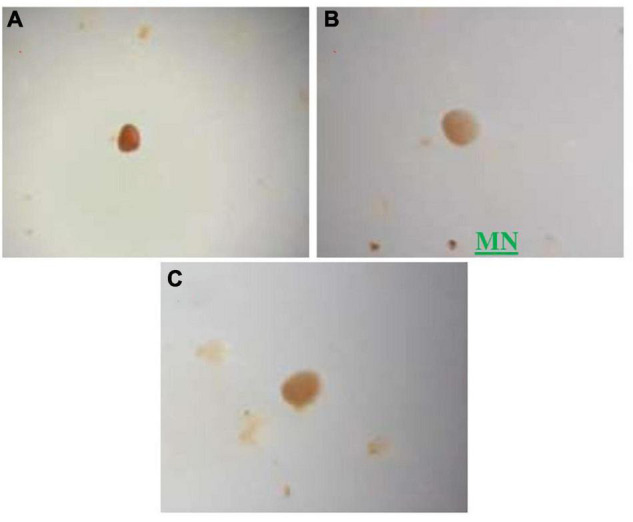
Some spores of different sizes and colors isolated from the phosphate rock, observations under the magnifying glass (GX40), **(A)** Morphotype 1, **(B)** Morphotype 2 named MN (native mycorrhizae used in our experiments), **(C)** morphotype 3.

**FIGURE 4:**
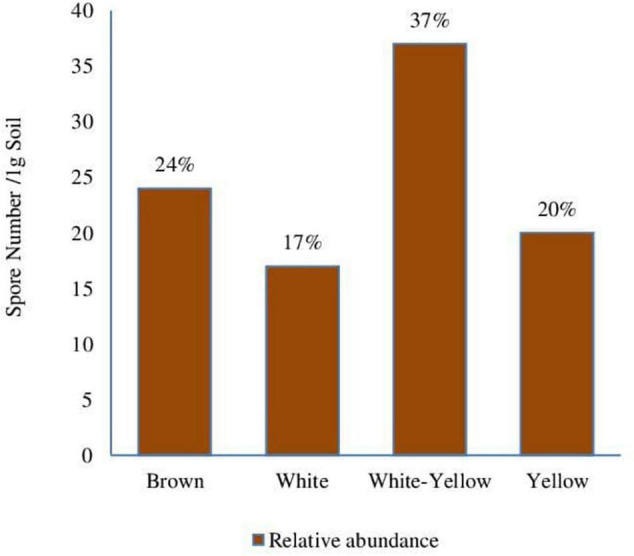
Relative abundance (%) of spore morphotypes in phosphate mine soil.

The presence of mycorrhiza in phosphate mine was very remarkable in this study, since it is an extreme environment, and phosphate inhibits mycorrhization in general. The sieving and decanting technique revealed the presence of less diversity in rock phosphate samples than in agricultural soil, as expected. The isolation of the spores from the phosphate mine samples showed the presence of four morphotypes (depending on the color criterion): brown, yellow, white-yellow, and white, with different sizes (Figures 3, 4). The density of spores was very low (103 spores/100 g of sample 10-fold less abundant than in agricultural soil samples).

Spores of the morphotype (white-yellow) were most abundant in phosphate mine with 39%. Then, the brown and yellow spores with 23 and 20%, respectively (Figure 4); finally, the white spores with relative abundance of 17%. The presence of mycorrhizal spores in this environment could result from an adaptation of some native plant species in the sampling area of phosphate mine soils. In order to assess this hypothesis, the analysis of mycorrhization of some native plants was performed.

#### Description of Mycorrhization in Wild Plants of Phosphate Mine

**FIGURE 5:**
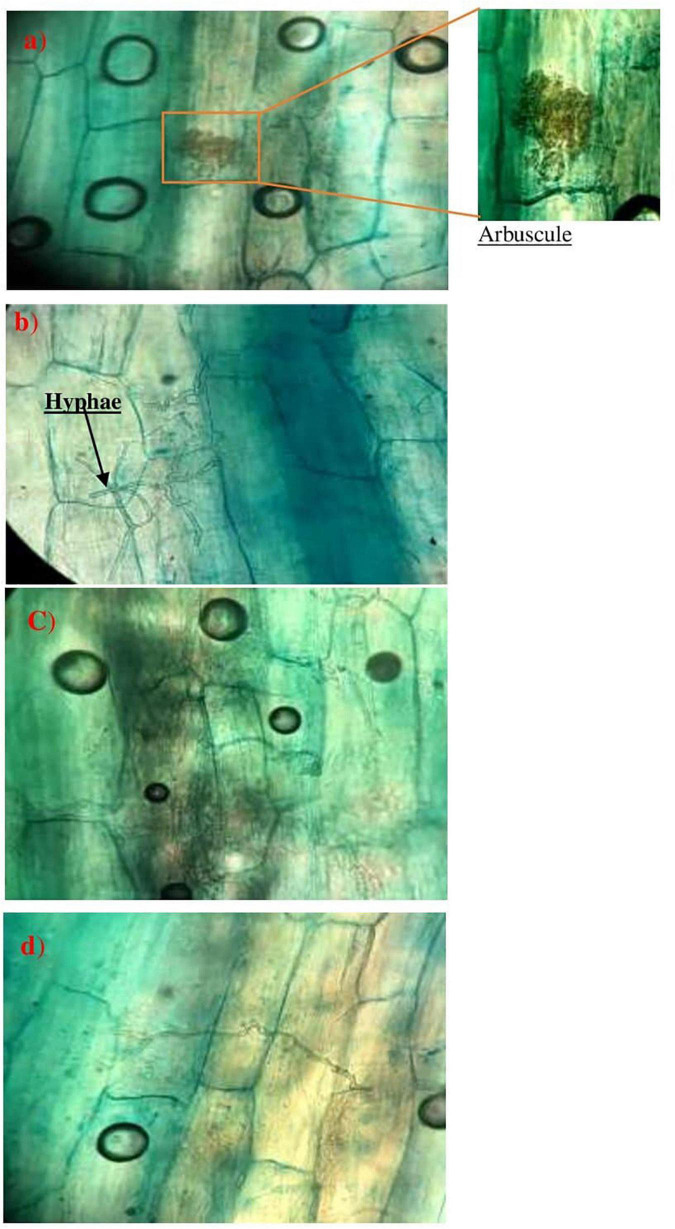
Observations of mycorrhizal structures in *Disitaria sanguinalis* **(a,b)** and *Alternanthera caracasana* **(c,d)**. wild plants in phosphate mines (×400).

Five wild plants species grown in phosphate mine were investigated for possible mycorrhization symbiosis. The obtained results showed that three of the five native plants showed a mycorrhizal infection (*Digitariasanguinalis*, *Solanum americanum*, and *Alternanthera caracasana*), with the presence of the penetration hyphae, appressoria, and arbuscules (Table 3 and Figure 5).

**TABLE 3:** Mycorhizzal colonization in wild plants of phosphate mine *Digitaria sanguinalis*; *Alternanthera caracasana* and *Solanum americanum.*

		Wild plants Species		
		*Digitaria sanguinalis*	*Alternanthera caracasana*	*Solanum americanum*
Colonisation	Mycorrhization frequency (F%)	46^a^	30^b^	22^c^
	Mycorrhization intensity (M%)	48^a^	26^b^	18^c^
	Richness on arbusculars (A%)	20^a^	8^b^	Absent

*The values indicated by different letters are statistically significant (P ≤ 0.05). The significations are made according to the application of different inoculations for the same parameter.*

The three studied plants: *Digitariasanguinalis*, *Alternanthera caracasana*, and *Solanum americanum* grow wildly in the phosphate mine area mainly during the humid season. They showed different rates of mycorrhization (Table 3). The mycorrhizal frequency (F%) was significantly higher in *Digitariasanguinalis* compared to the two other species, with 48% of mycorrhizal intensity and 20% of the richness in arbuscules (Table 3). This result was supported by the microscopic observation made in the roots section (Figure 5).

### Effect of Mycorrhization on Wheat Plant Growth, Nutrition, and Physiological Parameters

#### Description of Mycorrhization in Wheat Plants

All the morphotypes isolated and selected for this study, as previously described, were multiplicated in barley plants, in the aim to use barely infected roots as inoculum for wheat plants and to evaluate the effect of the isolated spore morphotypes on the nutritional profile, growth, and some physiological and biochemical parameters. The mycorrhizal colonization of wheat plants grown was estimated. Results of the mycorrhizal frequency (F%), the mycorrhizal intensity (M%), and the richness in arbuscules (A%) showed a high mycorrhization frequency in the plant inoculated with the *Glomus* sp2, MN, and by the positive control (inoculated by *Glomus intraradices*) than the plants inoculated with other two morphotypes (*Glomus* sp 1 and *Acaulosporalongula*) (Table 4). Significant differences in colonization frequency (F%), mycorrhizal intensity (M%), and richness on arbuscular (A%) according to the type of the spores used as shown in Table 4.

**TABLE 4:** Estimation of mycorrhizal infection in wheat plants inoculated with the 4 isolated morphotypes and the reference *Glomus intraradices*.

		*Glomus sp1*	*Glomus sp 2*	*Acaulospora longula*	*Morphotype MN*	*Glomus intraradices*
*Mycorrhizal infection indices*	Mycorrhization frequency (F%)	61^a^	74^b^	63^a^	80^b^	78^b^
	Mycorrhization intensity (M%)	41^a^	58^b^	48^a^	62^b^	54^b^
	Richness on arbuscular (A%)	22^a^	38^b^	19^a^	42^b^	40^b^

*The values indicated by different letters are statistically significant (P ≤ 0.05). The significations are made according to the application of different inoculations for the same parameter.*

**FIGURE 6:**
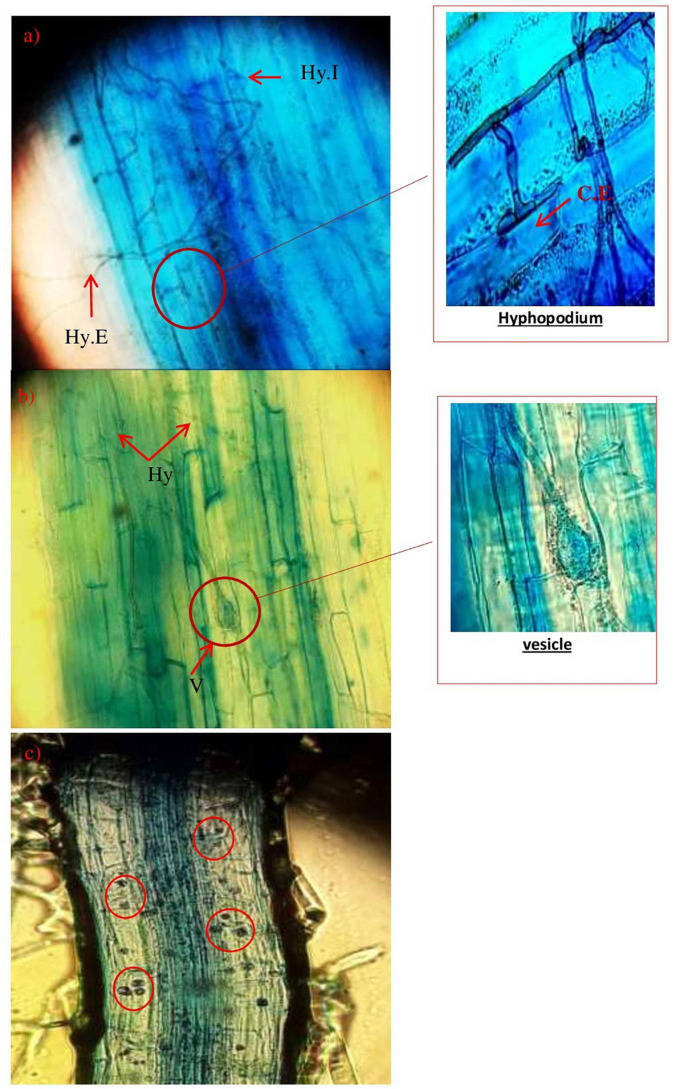
Evolution of AMF structure, hyphae, as well as the presence of a hyphopodium in wheat plants (Hy.E: extra root hyphae; Hy.I: intra-root hypha; CE: epidermal cell; V: vesicle) (×400).

Furthermore, Figure 6 shows the evolution of the mycorrhizal association according to the growth phases and phenology of the wheat plants after the inoculation by the morphotypes already selected and produced *Glomus* sp.1, *Glomus* sp.2, *Acaulosporalongula*, and morphotype MN. The formation of a hyphopod (Figure 6a) on the surface of some cells of the epidermis was observed. Already known as a pre-penetration device (PPD), a hyphopod guides the development of the fungus through the different layers of cells to the cells of the internal cortex where the arbuscules are placed and/or exchanges take place. Then, the fungus can finish its development cycle and form a new generation of spores.

Figure 6b shows the presence of a symbiotic structure (vesicle) since a root is considered to be mycorrhized when it has arbuscules and/or vesicles.

#### Impact of Different Mycorrhization Morphotype and Phosphate Nutritions on Wheat Plant Growth

**FIGURE 7:**
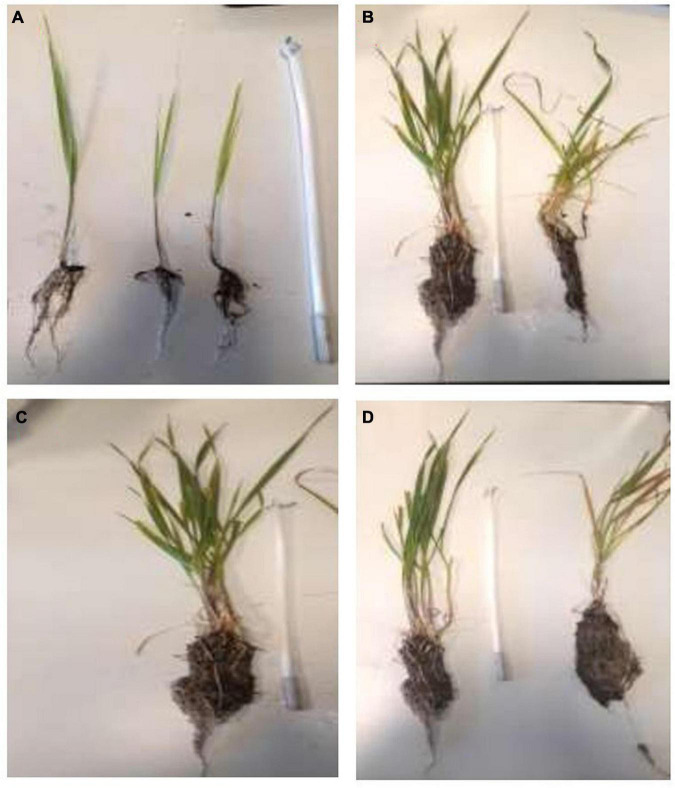
Variation of the growth in the presence and absence of mycorrhizal inoculation, with the presence of RP and TSP: **(A)** non-mycorrhizal plants; **(B)** mycorrhizae + TSP and mycorrhizae without (TSP and RP); **(C)** mycorrhizae + TSP; **(D)** mycorrhizae + TSP and mycorrhizae + RP (inoculum used: *Glomus* sp2).

Wheat plants were significantly influenced by the presence of mycorrhizae, also by phosphate fertilization sources (TSP vs. RP). All the mycorrhizae phenotypes significantly increased the growth of wheat plant shoots and roots as presented in Table 5. This growth improvement was largely driven by inoculation and nutrition, with maximum growth recorded in mycorrhized plants, in the presence of TSP and also RP (Table 5 and Figures 7B,C). In the absence of mycorrhization, the source of phosphate nutrition showed positive effect on the wheat growth, with a maximum reach with TSP and about 50% more growth than non-mycorrhized plants, without RP and TSP (Table 5 and Figure 7A). However, the presence of the different mycorrhizae inocula without any source of phosphate nutrition revealed the capacity of the *Glomus* sp2 to improve the plants growth, which reaches nearly the same values as the reference *Glomus intraradices* (Table 5). In the same sense, the inoculation with *Glomus* sp2 showed an increase of 73% in plant biomass than the non-mycorrhized plant with the presence of TSP in the medium (Figure 7C). Similarly, the *Glomus* sp2 inoculum resulted in a significant increase of plant growth under rock phosphate fertilization, which revealed good exploitation of this source as phosphate fertilization with mycorrhization (Figure 7D). However, the treatment MN + RP, the mycorrhizae spore isolated from the phosphate mine showed maximum growth of wheat plants with an increase of 90% compared to *Glomus* sp1. We noticed that this inoculum was isolated from the phosphate mine, an environment that presents a high phosphate concentration, which could explain this adaptation to RP (Table 5).

**TABLE 5:** Total growth and weight variation in wheat plant according to the presence of different MF inoculation, and the presence of TSP&RP in the medium.

Treatement	Length (cm)			Freash weight (g)		
	Total	Shoot	Root	Total	Shoot	Root
NM-(RP&TSP)	22 ± 3^a^	14 ± 2.2^a^	8 ± 1.5^a^**	4.5 ± 0.4^a^	2.8 ± 0.3^a^*	1.7 ± 0.4^a^**
NM+RP	25 ± 3.2^a^	16 ± 1.7^a^*	9 ± 1.6^a^**	5.2 ± 0.5^b^	3.4 ± 0.4^b^*	1.8 ± 0.3^a^**
NM+TSP	34 ± 4^b^	24 ± 2.8^b^*	10 ± 1.8^b^**	5.8 ± 0.6^b^	3.6 ± 0.3^b^*	2 ± 0.4^a^**
*G.sp1*-(RP&TSP)	26 ± 3^a^	17 ± 2^a^*	9 ± 1.2^a^**	5.8 ± 0.4^a^	3.1 ± 0.3^a^*	2.7 ± 0.2^a^**
*G.sp2*-(RP&TSP)	38 ± 3.2^b^	24 ± 2.8^b^*	14 ± 1.6^b^**	7 ± 0.5^b^	3.8 ± 0.4^b^*	3.2 ± 0.3^b^**
*A.long*-(RP&TSP)	28 ± 2.8^a^	17 ± 2^a^*	11 ± 1.7^a^**	6.1 ± 0.4^a^	3.2 ± 0.5^a^*	2.9 ± 0.4^a^**
MN-(RP&TSP)	31 ± 3^a^	20 ± 2.3^a^*	11 ± 1.4^a^**	5.9 ± 0.6^a^	3.2 ± 0.4^a^*	2.7 ± 0.3^a^**
MG-(RP&TSP)	40 ± 3.6^b^	28 ± 2.1^b^*	20 ± 2^c^	6.8 ± 0.3^b^	3.7 ± 0.3^b^*	3.1 ± 0.2^b^**
*G.sp1*+TSP	41 ± 2.6^a^	25 ± 2.2^a^*	16 ± 1.8^a^**	6,6 ± 0.5^a^	3,6 ± 0,2^a^*	3 ± 0.3^a^**
*G.sp2*+TSP	58 ± 6^b^	34 ± 3^b^*	24 ± 2^b^**	7.8 ± 0.6^b^	4.6 ± 0.3^b^*	3.2 ± 0.4^b^*
A.long+TSP	38 ± 2.6^a^	24 ± 2.2^a^*	14 ± 1.8^a^**	6.9 ± 0.3^a^	3.7 ± 0.2^a^*	3.2 ± 0.3^a^*
MN+TSP	43 ± 4^c^	27 ± 2.4^c^*	16 ± 1.8^c^**	8.2 ± 0.4^b^	4.5 ± 0.5^b^*	3.7 ± 0.4^b^*
MG+TSP	55 ± 5^b^	32 ± 2.4^b^*	22 ± 2^b^**	7.6 ± 0.5^b^	4.5 ± 0.4^b^*	3.1 ± 0.5^b^**
*G.sp1*+RP	28 ± 2.8^a^	18 ± 2^a^*	10 ± 2^a^**	6.2 ± 0.3^a^	3.3 ± 0.4^a^*	2.9 ± 0.4^a^*
*G.sp2*+RP	41 ± 4^b^	26 ± 2.4^b^*	15 ± 1.8^b^**	7.3 ± 0.5^b^	3.8 ± 0.4^b^*	3.5 ± 0.5^ab^*
*A.long*+RP	30 ± 2.6^a^	19 ± 2.2^a^*	11 ± 1.8^a^**	6.5 ± 0.3^a^*	3.5 ± 0.4^ab^*	3 ± 0.3^a^*
MN+RP	51 ± 5^a^	31 ± 2.6^a^*	20 ± 2^a^**	7.8 ± 0.5^b^	4.4 ± 0.4^b^*	3.4 ± 0.3^b^**
MG+RP	38 ± 4.5^b^	24 ± 2.8^b^*	14 ± 1.9^b^**	7.1 ± 0,5^b^	3.9 ± 0.3^b^*	3.2 ± 0.4^ab^*

*The values followed by different letters and asterisk are significantly different (P = 0.05). NM, non mycorrhized plants; G.sp1, Glomus sp1; G.sp2, Glomus sp2; A.long, Acaulospora longula; MN, morphotype isolated from rock phosphate sample; MG, Glomus intraradicess.*

The weights of wheat plants fit with the growth parameters, and they were significantly influenced by the presence of mycorrhizae, and also by phosphate fertilization sources. In the non-mycorrhized plants, the presence of RP and the TSP in the medium showed a significant positive effect on weight plants (Table 5). However, the presence of the different mycorrhizae inocula without any source of phosphate nutrition revealed the capacity of all the morphotypes to increase the weight of different parts of the plant compared to the non-mycorrhized plants, with the maximum increase shown in the *Glomus* sp2, which reach nearly the same values as the reference inoculum *Glomus intraradices* (Table 5). In the same sense, the inoculation with different morphotypes with the presence of RP and TSP in the medium reveals that *Glomus* sp2 showed an increase of 34% in plant biomass than the non-mycorrhized plant with the presence of TSP in the medium. Similarly, the *Glomus* sp2 inoculum showed a result in a significant increase of plant weight under rock phosphate fertilization, which revealed good exploitation of this source as phosphate fertilization with mycorrhization. However, the treatment MN + RP, the mycorrhizae spore isolated from the phosphate mine, showed maximum growth of wheat plants with an increase of 50% compared to NM + RP. We noticed that this inoculum was isolated from the phosphate mine, an environment that presents a high phosphate concentration, which could explain this adaptation to RP (Table 5).

#### Effect of Different Mycorrhization Morphotype and Phosphate Nutritions on Chlorophyll Content on Wheat Plants

**FIGURE 8:**
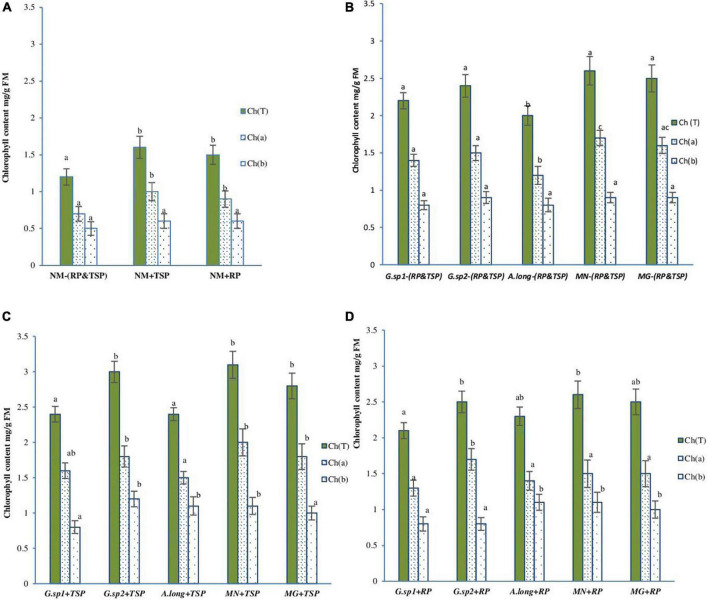
Chlorophyll (*a*,*b* and total) content variation in wheat plant with the presence of AMF, and TSP & RP in the medium: **(A)** non-mycorrhized plants (NM), **(B)** mycorrhized plants with the four inoculum and the absence of any phosphate sources M-TSP, -RP, **(C)** mycorrhized plants with the four morphotype with the presence of TSP free phosphate sources (M + TSP), **(D)** mycorrhized plants with the four morphotype with the presence of RP phosphate sources M + RP. The values followed by different letters are significantly different (*P* = 0.05).

The estimated levels of chlorophyll pigments showed that, in the absence of mycorrhization (NM), the content of total chlorophyll (Ch a + Ch b) is significantly higher (10%) in the presence of phosphate rock and TSP in the medium and reched 2.36 mg/g fresh matter (FM) (Figures 8C,D). However, the inoculation with the four morphotypes showed positive effects on chlorophyll content of wheat leaves, even in the absence of RP and TSP. The MN mycorrhized plants showed the maximum content with an increase of 116% compared to NM-RP and TSP (NM-RP, NM-TSP), followed by *Glomus* sp2 and the positive control inoculum (*Glomus intraradices*) by 98 and 100%, respectively (Figure 8B).

The presence of TSP in the medium (Figure 8C) increased the chlorophyll content, with the maximum value recorded in MN + TSP, *Glomus* sp2 + TSP, and the positive control inoculum (*Glomusintraradices*). This improvement reached a value of 158% for MN (spores isolated from phosphate mine) compared to NM-RP, -TSP, and 19% with MN-RP and TSP.

This content is slightly reduced in the presence of RP in the medium compared to the TSP; however, it was still much higher to the mycorrhized plant without RP and TSP (MN-RP, -TSP), and the non-mycorrhized plant. The inocula acted differently according to the presence of RP in the medium (Figure 8D). The inoculum isolated from the phosphate mine medium presented a good adaptation to rock phosphate fertilization regime, and helped the plant in physiological activity while *Glomus* sp1 and the *Acaulosporalongula* did not show a pronounced effect.

#### Effect of Different Mycorrhization Morphotype and Phosphate Nutritions on Total Phenolic Compounds Contents

**FIGURE 9:**
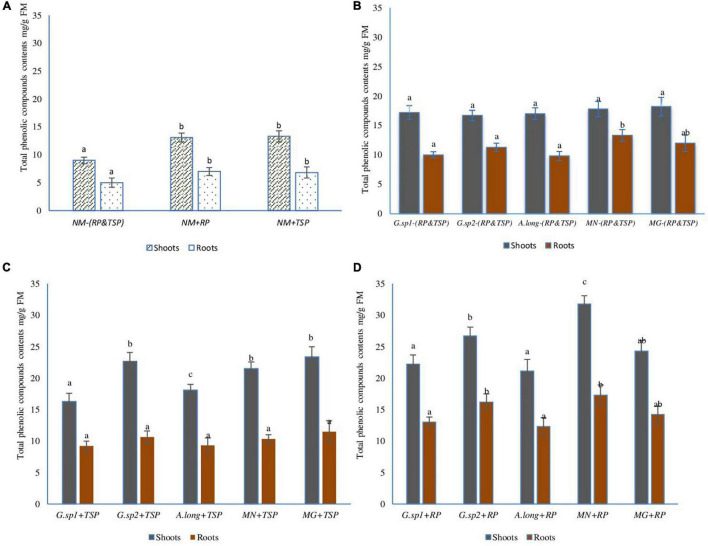
Total phenolic compound content in wheat plant according to the presence of AMF, and the presence of TSP & RP in the medium: **(A)** non-mycorrhized plants, **(B)** mycorrhized plants with the four inoculum and the absence of any phosphate sources, **(C)** mycorrhized plants with the four morphotype with the presence of TSP like free phosphate sources, **(D)** mycorrhized plants with the four morphotype with the presence of RP like phosphate sources. The values followed by different letters are significantly different (*P* = 0.05).

Total phenolic compounds (TPC) content changed with the presence of RP and TSP and, also, with mycorrhizae inocula (Figure 9). In the absence of mycorrhizae, the contents of TPC in both aerial and root parts of the plant increased significantly with the presence of RP and TSP (NM + TSP and NM + RP) in the medium (Figure 9A). However, in mycorrhizal plants with the absence of all sources of phosphate (Figure 9B), the TPC content increased very significantly with 21% higher TPC content than non-mycorrhized plants.

Inoculation with mycorrhizae and additions of TSP or RP improved the TPC content in both parts of the plant, with maximum increase recorded in the plant inoculated with *Glomus* sp2 and positive control inoculum MG, with 64% of increase compared to non-mycorrhized plants with the presence of TSP (NM + TSP), and 35% on mycorrhized plants without TSP. In parallel, some inocula (MN + TSP) showed higher results than others regarding the TPC content (Figure 9C).

The presence of RP in the medium impacted in a spectacular way the TPC content, which reached 128% in mycorrhized plants compared to non-mycorrhized plants with RP (NM + RP), and 100% compared to mycorrhized plants without any source of P. These interesting results recorded in plants were inoculated with the morphotype MN isolated from phosphate mine. In fact, this mycorrhizae helped the plant to use this source of phosphate efficiently. However, the other inocula acted differently but still showed increased levels of TPC.

## Discussion

The AMF present one of the preferential microorganisms for supplying the phosphate to the plants (Damin et al., 2020; Ranjbar Sistani et al., 2020). In this work, we reported, for the first time, the isolation of AMF from phosphate mine in the vicinity of wild plants that grow in this particular environment. According to many scientists, the high amount of phosphate inhibits the multiplication and the association of mycorrhizal spores with the plant (Sabannavar and Lakshman, 2009). In addition, the studied phosphate mine is located in a harsh environment characterized by water scarcity, high temperatures fluctuation, lack of precipitations, and minimal content of organic compounds. This environment is particualrly inadequate to the plant growth. AMF spores isolated from rock phosphate showed a high ability to improve plant growth in the presence of RP, more efficient than spores isolated from normal agricultural soil.

The deleterious impact of phosphate mine on plant growth and distribution was investigated along a decade (Sylvia and Neal, 1990). According to this author, phosphate mining operations can reduce population densities of VAM fungi in soil by destroying plant communities and by mixing top soil with both sub-soil and mine-soil materials. To overcome this delicate situation, the author tried to inoculate the native plants with vesicular-arbuscular mycorrhizal fungi (e.g., *Glomus etunicatum* and *Glomus intraradices*) for phosphate mine land reclamation. The native plants used for the revegetation of phosphate mine lands in Florida were *Aronia arbutifolia* (L.) Ell. (red chokeberry), *Clethra alnifolia* L. (sweet pepperbush), *Cornusfoemina Mill*. (swamp dogwood), *Ilex glabra* (L.) *Gray* (gallberry), and *Viburnum nudum* L.

The results obtained in this work showed less plant diversity in the Moroccan phosphate mine samples. Three plant species; *Digitariasanguinalis*, *Alternanthera caracasana*, and *Conyza canadensis* that are native in this environment presented mycorrhizal richness. However, this richeness was significantly lower than that reported in agricultural soil. These species could be good candidates for indigenous spore multiplication. In fact, the phosphate mine is a very challenging environment for plant mycorrhization, not just because of the presence of inorganic phosphate but also the existence of the high rate of metals, which could explain the less abundance of wild-plant species (Mabrouk et al., 2020) and poor vegetation. Similar results were found in the Metlaoui phosphate mining basin (Gafsa, Tunisia), where only two native plants, *Moricandia arvensis* and *Diplotaxis harra* (*Brassicaceae*), were able to tolerate high concentrations of Cd, Ni, Zn, and Cr (Mabrouk et al., 2020).

The second factor that could explain the limited plant diversity and AMF in this environment arises from the allelopathic response of native plants against other weeds. For example, *Digitariasanguinalis* has a great ability to inhibit the growth of other plant species *via* allelopathic response. This plant exhibits its harmful allelopathic effects on crop species through different mechanisms, like phenolic acid compounds (Rashidi et al., 2020). This ability enhanced significantly with the presence of AMF. According to Rashidi et al. (2020), the inoculation of *Digitariasanguinalis* and *S. nigrum* plants with AMF showed greater competitive ability and allelopathic potential of this weed when associated with AMF, which makes it a good competitor against other plant species in the environment. According to Rashidi et al. (2020), this native plant seems to be more adapted to made association with mycorrhizae, especially with *Funneliformismosseae*, *Rhizoglomusfasciculatum*, and *Rhizoglomusintraradices*.

The poor abundance of host plants in the phosphate mine area impacted negatively the AMF diversity, which explains the abundance and diversity of AMF recorded in our study (Jan et al., 2006; Rosendahl and Matzen, 2008). Moreover, the chemical fertilizer (especially phosphorus) can decrease AMF root colonization and could have negative impact on AMF communities (Entry et al., 2002; Tang et al., 2005).

According to Xiao C. Q. et al. (2009), the plant species grown in the phosphate mine were more adapted to high phosphate concentration. In China, 38 plant species belonging to 7 families were collected from the phosphate mining areas. However, only 12 plant species were shown to have the ability to accumulate phosphorus. The two species, which have been more adapted, *Pilea sinofasciata* and *Polygonum*, could accumulate, respectively, 3.4 and 7 times phosphorus higher than in the non-mining areas.

One of the native plants investigated in this work (*Digitariasanguinalis*) has a great ability to establish association with mycorrhizae, especially *Funneliformismosseae*, *Rhizoglomusfasciculatum*, and *Rhizoglomusintraradices* (Xiao G. et al., 2009). According to Rashidi et al. (2020), this association improved the ability of *Digitariasanguinalis* to adapt to the harsh conditions by increasing the photosynthetic rate and biomass (from 26 to 49%). However, sometimes, the association could be ineffective or non-functional, which explains the non-adaptation of other species in the phosphate mine area.

In terms of spores diversity, according to Maazouzi et al. (2020), the Rhamna region holds great diversity of AMF spores, divided into three orders (*Glomerales*, *Diversisporales*, and *Archaeosporales*), and many Genus, *Glomus* (10 species), *Rhizophagus* (2 species), *Funneliformis* (4 species), *Claroideoglomus* (1 species), *Dentiscutata* (2 species), *Gigaspora* (3 species), *Scutellospora* (2 species), *Racocetra* (1 species), *Acaulospora* (6 species), *Entrophospora* (1 species), *Diversispora* (1 species), and *Ambispora* (1 species). These genera seem to be highly adapted to the host plants of the region but, of course, differ from one site to another one according to the above-cited parameters.

The native plants of the Rhamna region presented different rates of mycorrhization, which vary between 83.33 and 76.67% in *Acacia gummifera* and *Acacia* sp., respectively (Maazouzi et al., 2020), However, according to the same study, 50–60% of *Acacia aneura* and *Prosopis juliflora* roots were mycorrhized. This difference on mycorrhization frequency is probably due to soil composition, environmental conditions, and plants hots (Vivekanandan and Fixen, 1991; Hazzoumi et al., 2017; Maazouzi et al., 2020).

The main conclusion about the plants in phosphate mine and their associated AMF spores could be summarized as follows: the native plants growing in phosphate mine could be adapted to high concentrations of phosphate and heavy metals. In order to tolerate this environment, plants growing in this area develop great ability to make an association with AMF. This later helps the plants to persist and resist. The interaction plant AMF leads to an allelopathic response, which limits the growth of other plants. Simultaneously, the less abundance of plant hosts limits AMF spores multiplication and limits the AMF genera existing in this particular environment (Hazzoumi et al., 2017; Maazouzi et al., 2020).

Regarding inoculation assays in wheat plants, we can confirm that the presence of RP and TSP in a medium impacted significantly the plant growth; this source of phosphate nutrition was exploited differently according to the presence of the AMF inoculum or not, and also according to the type of inoculum. We previously reported that the mycorrhized plants presented a high rate of growth in both parts (the areal part and roots) (Hazzoumi et al., 2015), which is confirmed in other plants (*ocimumbasilicum*) (Ferahani et al., 2008; Zolfaghari et al., 2013). These studies showed that the inoculation of basil plants by *G. intraradices* and some indigenous MF enhanced the growth of both parts of the plants (82% for the areal part and more than 52% for the roots). Similar results were reported in other species, such as *Lactuca sativa* L., *Lonicera confusa*, and *Vicia faba* L. Omar (1998) recorded an increase in wheat plant growth after inoculation with *G. constrictum*. According to the same study, the greatest positive effect on growth was recorded after using a mixture *of G. constrictum* and RP, which increased phosphorus nutrition. Wang et al. (2020) showed that phosphorus nutrition increased the wheat plant growth, especially in the presence of AMF, which increased the nutrient absorption and, also, the resistance against insects and pathogens. Some studies reported the positive effects of AMF and RP association on plant growth, and explained how the fungi can lead to better exploitation of this phosphate source. Osorio and Habte (2013) showed that the AMF *Glomus fistulosum* had a positive effect on the growth and the nutritional profile of L*eucaena* plants in the presence of RP; however, the best efficient results were recorded in experiments using combination *of G. fistulosum* and phosphate-solubilizing fungi (*Mortierella* sp.). These results were confirmed by Omar (1998), who found that the better exploitation of RP was recorded with a mixture of inoculum of AMF and phosphate-solubilizing fungi (*Aspergillus niger* and *Penicillium citrinum*).

The results of the positive enhancement in morphologic parameters are supported by the physiological essays, especially the photosynthetic activity, which was impacted by the presence of RP and TSP in the medium and, also, the presence of AMF. The results of this study showed that the presence of RP and TSP in the medium increased the chlorophyll content of wheat leaves, especially with the inoculation of AMF spores. Similar effects were previously reported by Hazzoumi et al. (2015), which recorded a high chlorophyll content in basil plants inoculated by *G. intraradices* (more than 12%). This increase was higher under stress conditions (e.g., water stress) where the mycorrhization increased chlorophyll content of plants with more than 64%. According to the same authors, the nutritional profile (especially the phosphorus nutrition) was increased. The same results were confirmed by Rashidi et al. (2020) *in Digitariasanguinalis*, showing that inoculation with *Funneliformismosseae*, *Rhizoglomusfasciculatum*, and *Rhizoglomusintraradices* resulted in about 50% more biomass than uninoculated plants.

The mycorrhization leads to better exploitation and absorption of the nutrients, which explained the increase of chlorophyll content. At the same time, it provides to the plant good water exploitation (the main parameters that induce an alteration of the chlorophyll synthesis) (Hazzoumi et al., 2015). The combination of mycorrhizae and a phosphate source leads to a better chlorophyll synthesis, which is demonstrated by enhancements in the morphologic parameters.

The difference in chlorophyll content reported in plants inoculated by different spores could be explained by the rate of mycorrhization, as shown in Table 4, because a high rate of mycorrhization and arbuscule (A% = 42%) (an interface of nutrition change between plants and AMF). This implied better exploitation of nutrients and water and, also, plant protection. Moreover, there is an affinity between the inoculum and plants, which depend on medium composition. Indeed, plants inoculated with the spores from the phosphate mine environment seem more adapted when the RP is used as fertilizer. In the same sense, a better nutrition profile (TSP) with the presence of AMF led to a greater rate of chlorophyll content (the maximum value recorded in MN + TSP, *Glomus* sp2 + TSP), with content varies in the average of 3 mg/g FM (Figure 8).

Our results are in accordance with Aliferis et al. (2015) and Sunitha et al. (2018), who confirmed that AMF increased the chlorophyll synthesis in *Salix purpurea* L., Salicales: (Salicaceae) plants and positively impacted other physiological parameters. Levy and Krikun (1980), Allen et al. (1981), Kucey and Paul (1982), and Snellgrove et al. (1986) also confirmed these findings and demonstrated that mycorrhizae stimulated photosynthetic activities in many species. They reported that this photosynthesis improvement was due to the increase of the P nutrition, which is necessary for the photosynthetic reaction (the assimilation of CO_2_ by the stomata). This stimulation could be linked also to an increase in the leaf area and the hydric status on the leaves (Snellgrove et al., 1982; Fredeen and Terry, 1988; Hazzoumi et al., 2015).

Total phenolic compounds content changed significantly according to the presence or the absence of AMF and, also, according to the presence of RP and TSP in the medium. Generally, the mycorrhization pushed the plant to synthesize phenolic compounds as natural response to the association plant/AMF. However, after the first phase of the association, we can record diminution of the TPC level if the plant is in an optimum medium of growth, the presence of RP in the medium pushed the plant to keep TPC synthesis, which explained this increase in TPC contents even in the absence of AMF inoculation (Hazzoumi et al., 2015). According to Khan et al. (2010), the P-solubilizing fungi produce more organic acids than bacteria and consequently exhibited greater P-solubilizing activity. These results are supported by the work of Wang et al. (2020) on wheat plants, which concluded that AMF increased the phenolic compounds level and contributed to plant defense against pathogens and, also, to P solubilization (Sunitha et al., 2018).

Generally, secondary metabolites are more influenced by a nutritional profile and soil microflora, and phenolic compounds are important secondary metabolites. The increased level of TPC in the roots is mainly explained by the direct contact of this part with RP and AMF. However, differences recorded on TPC content according to inoculation of different spores are mainly due to the mycorrhization level of each one and, also, to organic acid produced by the fungi and the plant for phosphate solubilization (Iker et al., 2004; Hazzoumi et al., 2015).

Wheat plants inoculated with the native spores of phosphate mine presented a high level of TPC synthesis, which allows the plant better exploitation of P existing in rock phosphate, as confirmed by the growth parameters and chlorophyll contents (Figure 8 and Table 5).

The soil pH in the non-mycorrhized plant is almost neutral, between 7 and 7.3, and we should mention that the RP slightly reduced the pH level (Table 6). In the mycorrhized soil without RP and TSP, we recorded a pH drop; however, the addition of TSP and RP on the medium leads to a significant reduction of the pH level, with the most reduction recorded in the plant inoculated with the MN spores with the RP (pH = 6.3) (Table 6).

**TABLE 6:** Change on the pH level according to the type of inoculum and also according to the existing of RP and TSP in the medium.

Treatments	NM-(RP&TSP)	NM+RP	NM+TSP		
pH	7.3	7	7.2		
Traitements	G.*sp1-(*RP&TSP)	G.*sp2*-(RP&TSP)	A.*long*-(RP&TSP)	MN-(RP&TSP)	MG-(RP&TSP)
pH	6.8	7	7	6.8	6.7
Traitements	G.*sp1*+TSP	G.*sp2*+TSP	A.*long* +TSP	MN+TSP	MG+TSP
pH	6.6	6.7	6.8	6.5	6.5
Traitements	G.*sp1*+RP	G.*sp2*+RP	A.*long* +RP	MN+RP	MG+RP
pH	6.6	6.8	6.5	6.3	6.5

This result supported our explanation about the impact of mycorrhizal fungi on the soil pH, as the first response to subtilize the phosphate rock. This diminution of the pH presented a mechanism that consists on the acidification of the medium, and many reactions of chelation and exchange in the rhizosphere environment (Cunningham and Kuiack, 1992). However, some fungi did not tolerate an acid medium, and, in the same time, they solubilized rock phosphate by some organic acid synthetized by the fungi in a tolerable amount (Omar, 1998).

In the study of Van Aarle et al. (2002), authors tested the response of arbuscular mycorrhizal fungi (AMF) *Scutellosporacalospora* and *Glomus intraradices* at different soil pHs (5 and 6), in association with *Plantago lanceolata*. Results showed that both fungi formed more extraradical mycelium at the higher pH, and both fungi formed more arbuscules and vesicles in low pH, with a high rate of colonization of plants by fungi.

**FIGURE 10:**
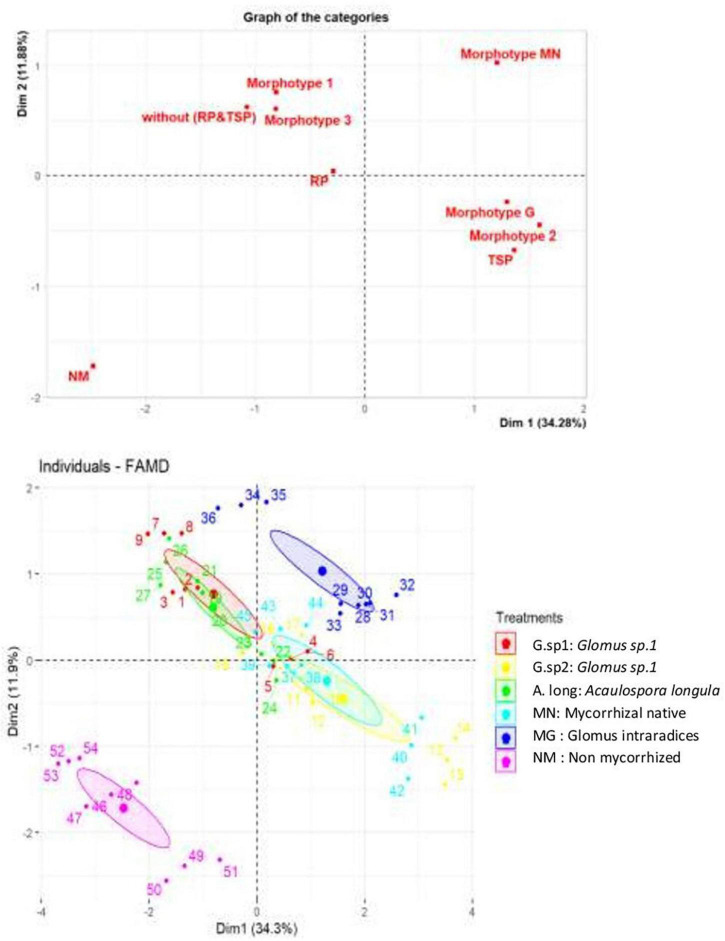
Principal component analysis of wheat plant according to the presence/absence of AMF, TSP & RP in the medium and also the impact of each inoculum and P source on chlorophyll content, stems, and roots growth.

The principal components analysis confirmed the previous results of growth and chlorophyll content, in relation with P sources and the AMF inoculation (Figure 10). The mycorrhization pulls the curve in the other sense than non-mycorrhized plants; in the same way, the presence of TSP and RP in the medium with the absence of P source. This means that the presence of AMF improved the chlorophyll content and the growth parameters; this positive impact was also influenced by the P sources (Figure 10A). However, the RP nutrition presented a very positive impact on all the parameters studied (chlorophyll content, growth of roots and stems), but with the inoculation of morphotypes able to explore this source of phosphate.

The AMF morphotypes affected positively the growth parameters and chlorophyll content. The inoculum isolated from the phosphate mine showed a positive correlation with all the parameters represented in the PCA contrariwise to the absence of the inoculum; however, *Glomus sp1* and *Acaulosporalongula* almost act in the same way, and the positive control *Glomusintraradices* and *Glomus* sp2 almost reflected a similar effect (Figure 10B).

## Conclusion

Boosting and improving plant growth and assimilation of nutriments present a crucial challenge in agriculture; in this vision, we put the light on the AMF isolated from agriculture soil and phosphate mine soil to improve thus parameters. The results showed high diversity of spores in the agricultural medium, in comparison to the phosphate mines area, with Glomus, Gigaspora, and Acaulospora present the main spores isolated.

The inoculation of wheat plants with AMF spores improved the growth of the plants *via* the increase of the root surface, which makes the plant able to explore a large surface of the soil and increases their nutritional profile, especially the spores isolated from phosphate mines, which present the results the more significant in the morphological and biochemical parameters (growth, chlorophyll phenolic compounds).

The results showed less diversity of AMF in the natural phosphate samples compared to soil samples.

Moreover, it is considered that there is an impact on the phosphate solubilization, which explained the positive results obtained in the mycorrhizal plant with rock phosphate (M + RP). Native plants and AMF from phosphate mines could present a reservoir of unexploited microbial diversity that could help in the agronomic efficiency of rock phosphate used as fertilizer.

## Data Availability Statement

The datasets presented in this study can be found in online repositories. The names of the repository/repositories and accession number(s) can be found in the article/supplementary material.

## Author Contributions

ZH, SA, NE, YZ, RD, BB, and IMK participated in designing the study, data processing, spores identification, performed the statistical analysis, and the draft of the manuscript. All authors read and approved the final manuscript.

## Conflict of Interest

YZ was employed by OCP Group. The remaining authors declare that the research was conducted in the absence of any commercial or financial relationships that could be construed as a potential conflict of interest.

## Publisher’s Note

All claims expressed in this article are solely those of the authors and do not necessarily represent those of their affiliated organizations, or those of the publisher, the editors and the reviewers. Any product that may be evaluated in this article, or claim that may be made by its manufacturer, is not guaranteed or endorsed by the publisher.
